# Morphological and molecular diversity patterns of the genus *Tropodiaptomus* Kiefer, 1932 (Copepoda, Calanoida, Diaptomidae) in Thailand

**DOI:** 10.1038/s41598-022-06295-4

**Published:** 2022-02-09

**Authors:** Thanida Saetang, Federico Marrone, Luca Vecchioni, Supiyanit Maiphae

**Affiliations:** 1 Department of Zoology, Faculty of Science, Kasetsart University, Bangkok, Thailand; 2 Department of Biological, Chemical and Pharmaceutical Sciences and Technologies, University of Palermo, Palermo, Italy

**Keywords:** Molecular biology, Zoology

## Abstract

*Tropodiaptomus* is one of the most specious genera in the family Diaptomidae, but it is often rare in terms of distribution and abundance. Moreover, *Tropodiaptomus* species show a noteworthy variability in some of the morphological characters considered of prime importance in diaptomid taxonomy, and the presence of cryptic or pseudocryptic species is likely. Thus, through a geographically-wide sampling in Thailand, we aimed to investigate the local diversity of the genus and to compare the morphological and molecular diversity pattern based on mitochondrial and nuclear genes sequences. DNA taxonomy was also implemented in order to check whether the *Tropodiaptomus* lineages were independent species according to the “evolutionary genetic species concept”. Six *Tropodiaptomus* morphospecies were found, three of which are putative species new to Science pending a formal description. The finding of such a high incidence of undescribed species stresses the existence of a significant “Linnean shortfall” affecting Thai diaptomids. The molecular results showed that most of the studied species could be identified consistently with their morphology-based taxonomy. However, *Tropodiaptomus vicinus* and *T*. cf. *lanaonus* showed a high level of genetic diversity, suggesting that traditional morphological techniques might be inadequate for correctly assessing their taxonomical status.

## Introduction

Taxonomic crypsis, i.e., the existence of distinct evolutionary entities which cannot be told apart based on morphology, is particularly frequent for those taxa where morphological conservatism or poorly understood morphological plasticity prevent from a morphology-based sound assessment of their actual diversity (e.g.^1^). Such a phenomenon is well known for copepods (e.g.^2^), and has been lately observed among several Holarctic diaptomid copepod genera (e.g.^3–7^). Conversely, no such evidence is to date available for diaptomids occurring in the Oriental region. Since cryptic and pseudocryptic species are assumed to be widely distributed in nature and among biogeographical regions^8^, and their discovery and description are pivotal to the correct assessment of actual biodiversity patterns^9^, we have investigated the morphological and genetic diversity patterns of the diverse copepod genus *Tropodiaptomus* Kiefer, 1932 in Thailand with the aim of contributing to a sounder assessment of the actual diaptomid diversity of Indo-Burma biodiversity hotspot.

With over 60 formally described species, mostly distributed around Africa and Asia^10,11^, *Tropodiaptomus* is the most speciose genus in the family Diaptomidae. To date, eight *Tropodiaptomus* species, i.e., *Tropodiaptomus doriai* (Richard, 1894), *T*. *hebereri* (Kiefer, 1930), *T. lanaonus* Kiefer, 1982, *T. megahyaline* Saetang, Sanoamuang & Maiphae, 2020, *T. oryzanus* Kiefer, 1937, *T*. *ruttneri* (Brehm, 1924), *T. vicinus* (Kiefer, 1930) and *Tropodiaptomus* sp., have been recorded from Thailand, where they occur in various types of habitats such as ponds, lakes, rivers, roadside canals, rice fields, and so forth (e.g.^12–14^, and references therein), being always rare both in term of distribution and abundance. Studies available to date for Asian *Tropodiaptomus* focused on morphological characters only. However, as already stressed by Lai & Fernando^15^, Lai et al.^16^, Defaye^17^ and Ambedkar^18^, *Tropodiaptomus* species show a noteworthy variation in the morphology of male fifth pair of legs and antennule, i.e., those characters which are traditionally used for species identification in diaptomid copepods (e.g.^13,19^). It is thus likely that the diversity of the genus is to date inadequately known, thus preventing the understanding of the distribution, ecology, and natural history of its species.

Based on an integrative approach including morphological and genetic data, we thus aim (i) to investigate the species diversity of the genus *Tropodiaptomus* in Thailand, (ii) to compare morphological and molecular diversity patterns based on mitochondrial and nuclear genes sequences, and (iii) to investigate the taxonomical value of the morphological characters currently used for species identification.

## Materials and methods

### Sample collection and morphological identification

**Figure 1:**
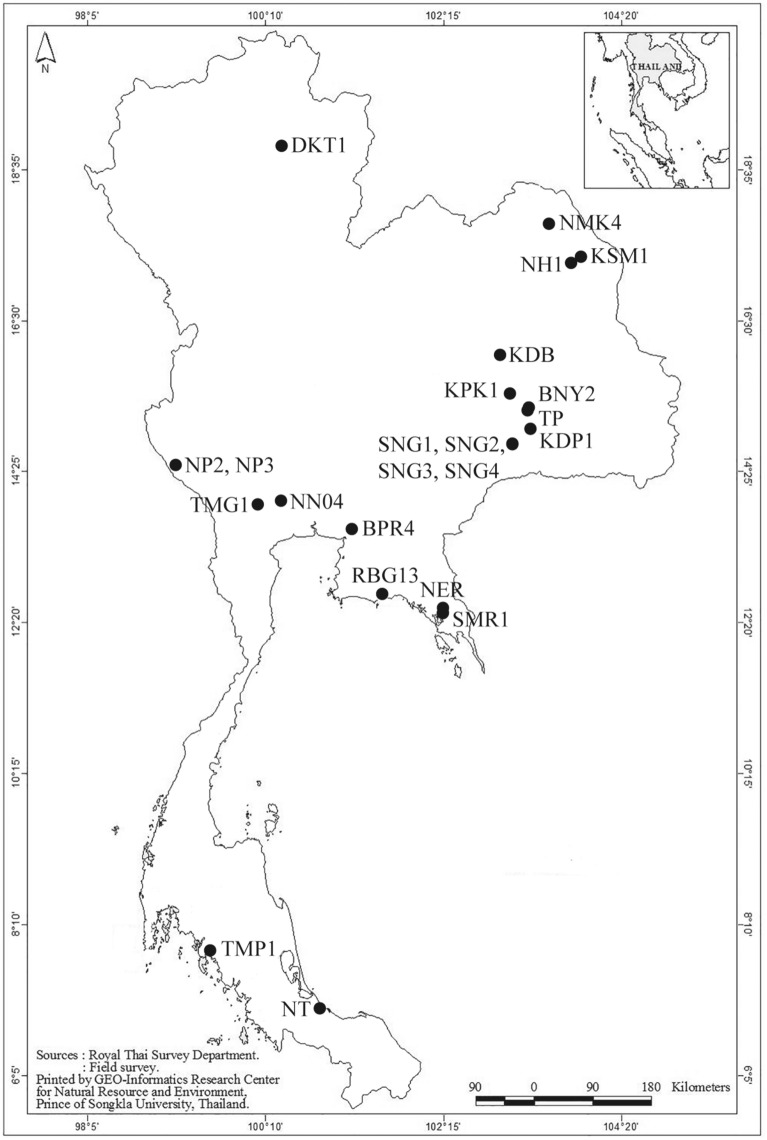
Sampling sites of the analyzed *Tropodiaptomus* samples. Refer to Table 1 for locality codes. (This map was created by GEO-Informatics Research Center for Natural Resource and Environment, Prince of Songkla University and modified by authors with Adobe Illustrator CS5).

Out of 468 zooplankton samples collected from 2017 to 2019 in 196 permanent and temporary water bodies throughout Thailand, 29 samples containing *Tropodiaptomus* spp. were found from 23 sites (Fig. 1, Table 1). Copepods were sampled with a 60 µm mesh-sized hand net, immediately preserved in 99% ethanol and kept in a cool box. *Tropodiaptomus* specimens were then sorted out in the laboratory under a stereo microscope, prepared according to Dussart and Defaye^20^, and identified to species level according to Lai et al.^16^, Lai and Fernando^21^, Kiefer^22^, Sanoamuang^23^ and Saetang et al.^13^. The morphology of 108 collected specimens was carefully checked and compared with that of the known species of the genus. Particular attention was paid to the ornamentation of the basis and second exopod segment of male right fifth pair of legs (P5) and of the exopod segment of male left P5, to the length of the spinous process occurring on the antepenultimate segment of adult male right antennule, and to the number of setae occurring on the 13^th^ segment of male left antennule. Collected samples are now stored at the Department of Zoology, Kesetsart University, Thailand.

**Table 1:** Synopsis of the *Tropodiaptomus* species investigated in the frame of present study, information about the sampling locality, and GenBank Accession Numbers, A.N.

Taxa	Sampling site	Type of habitat	Coordinates	Code of specimens	Sex	Sampling date	GenBank A. N		
							12S	28S	ITS2
*T. oryzanus*	Ban Na Yom, Surin, NE	Rice field, T	15°10′00.7″ N 103°48′54.1″ E	BNY2 166	F	03.06.2019	OL628744	–	OL527713
				BNY2 167	F	03.06.2019	OL628745	–	OL630131
				BNY2 203	F	03.06.2019	OL628746	–	OL527714
	Kud Pha Thai, Surin, NE	Swamp, T	14°55′17.5″ N 103°47′21.0″ E	KDP1 168	M	03.06.2019	OL628747	–	OL527715
	Huai Saneng, Surin, NE	Man-made pond, T	14°47′42.5″ N 103°28′30.4″ E	SNG4 158	M	03.06.2019	OL628748	OL527669	OL527716
				SNG4 185	M	03.06.2019	OL628749	OL527670	OL527717
				SNG4 278	M	03.06.2019	OL628750	–	OL527718
*T. vicinus*	Kae Dam Bridge, Mahasarakham, NE	Swamp with aquatic plants, T	16°01′26.9″ N 103°23′31.2″ E	KDB 31	M	25.05.2018	OL628699	–	OL584127
				KDB 188	M	04.06.2019	OL628700	OL584161	OL584128
				KDB 189	M	04.06.2019	OL628701	OL584162	OL584129
	Kud Pha Thai, Surin, NE	Swamp with aquatic plants, T	14°55′17.5″ N 103°47′21.0″ E	KDP1 34	M	26.10.2018	OL628702	–	OL584130
				KDP1 35	M	26.10.2018	OL628703	OL584163	OL584131
				KDP1 110	M	26.10.2018	OL628704	OL584164	OL584132
				KDP1 169	F	03.06.2019	OL628705	–	OL584133
	Khlong Phrai Kla, Surin, NE	Roadside canal with aquatic plants, P	15°20′38.2″ N 103°32′18.7″ E	KPK1 240	M	13.10.2017	OL628706	–	OL630116
				KPK1 242	M	13.10.2017	OL628707	OL584165	OL584134
				KPK1 306	M	13.10.2017	OL628708	–	OL584135
	Nong Han, Sakon Nakhon, NE	Lake with aquatic plants, P	17°15′25.9″ N 104°09′36.2″ E	NH1 171	F	27.10.2018	OL628709	–	OL584136
	Ban Na, Nakhon Nayok, C	Canal with aquatic plants, P	14°16′28.5″ N 101°02′53.1″ E	NN04 55	M	12.10.2018	OL628710	–	OL584137
				NN04 56	F	12.10.2018	OL628711	OL584166	OL584138
				NN04 57	F	12.10.2018	OL628712	–	OL584139
	Rayong botanic garden, Rayong, E	Peatswamp, T	12°39′06.8″ N 101°32′52.4″ E	RBG13 129	M	12.10.2017	OL628713	OL584167	OL584140
				RBG13 130	M	12.10.2017	OL628714	–	OL584141
	Sa Mer Rat swamp, Trat, E	Swamp with aquatic plants, P	12°28′04.0″ N 102°21′20.6″ E	SMR1 128	F	07.09.2017	OL628715	–	OL630117
				SMR1 206	M	07.09.2017	OL628716	OL584168	OL584142
	Huai Saneng, Surin, NE	Swamp with aquatic plants, P	14°48′22.4″ N 103°29′10.5″ E	SNG1 4	F	25.05.2018	OL628717	–	OL584143, OL584144
				SNG1 5	F	25.05.2018	OL628718	–	OL584145
				SNG1 104	M	25.05.2018	OL628719	–	OL584146
				SNG1 107	F	25.05.2018	OL628720	–	OL584147
		Swamp, T	14°48′44.2″ N 103°29′30.5″ E	SNG2 133	F	25.05.2018	OL628721	–	OL584148
				SNG2 134	F	25.05.2018	OL628722	–	OL584149, OL584150
		Swamp with aquatic plants, T	14°47′40.8″ N 103°28′29.5″ E	SNG3 11	F	13.10.2017	OL628723	–	OL584151, OL584152
				SNG3 305	M	03.06.2019	OL628724	–	OL584153, OL584154
	Tha Muang, Kanchanaburi, W	Man-made pond with aquatic plants, P	13°56′25.7″ N 99°38′26.0″ E	TMG1 33	F	17.10.2018	OL628725	–	OL584155
	Ta Ma Praw, Krabi, S	Swamp with aquatic plants, P	7°44′06.5″ N 99°10′37.9″ E	TMP1 123	F	12.11.2017	OL628726	–	OL584156
				TMP1 124	M	12.11.2017	OL628727	–	OL584157
	Ta Phet, Surin, NE	Swamp with aquatic plants, P	15°08′21.6″ N 103°48′50.5″ E	TP 178	F	26.10.2018	OL628728	OL584169	OL584160
				TP 225	M	13.10.2017	OL628729	–	OL584158
				TP 226	M	13.10.2017	OL628730	–	OL584159
*T.* cf. *lanaonus*	Bang Pakong, Chachoengsao, C	Rice field, T	13°35′55.0″ N 101°04′06.7″ E	BPR4 174	F	22.09.2018	OL628731	–	OL630118
	Khlong Phrai Kla, Surin, NE	Roadside canal with aquatic plants, T	15°20′38.2″ N 103°32′18.7″ E	KPK1 238	M	13.10.2017	OL628732	–	OL630119
				KPK1 239	M	13.10.2017	OL628733	OL584124	OL630120
				KPK1 241	M	13.10.2017	OL628734	–	OL630121
				KPK1 131	F	26.10.2018	OL628735	–	OL630122
				KPK1 132	F	26.10.2018	OL628736	–	OL630123
				KPK1 177	M	03.06.2019	OL628737	OL584125	OL630124
	Nong E-ruem, Trat, E	Swamp with aquatic plants, P	12°31′27.2″ N 102°20′12.6″ E	NER 53	M	07.09.2017	OL628738	–	OL630125
				NER 175	F	07.09.2017	OL628739	–	OL630126
				NER 176	F	07.09.2017	OL628740	–	OL630127
	Nong Mha Khao, Bueng Kan, NE	Rice field, T	17°49′23.5″ N 103°55′19.4″ E	NMK4 142	M	31.06.2019	OL628741	–	OL630128
	Na Thap, Songkhla, S	Swamp with aquatic plants, T	7°00′49.8″ N 100°41′44.5″ E	NT 173	F	21.06.2018	OL628742	–	OL630129
				NT 301	M	21.06.2018	OL628743	OL584126	OL630130
*Tropodiaptomus* sp.1	Dok Kham Tai, Phayao, N	Swamp, T	19°13′57.6″ N 100°02′56.5″ E	DKT 120	M	31.06.2019	OL628751	–	OL630132
				DKT 121	M	31.06.2019	OL628752	OL584120	OL630133
				DKT 138	M	31.06.2019	OL628753	OL584121	OL630134
				DKT 302	M	31.06.2019	OL628754	–	OL630135, OL630136
*Tropodiaptomus* sp.2	Nong Ping, Kanchanaburi, W	Swamp, T	14°38′49.1″ N 98°33′48.8″ E	NP2 154	M	22.06.2019	OL628755	–	OL630137
				NP2 200	M	22.06.2019	OL628756	OL584122	OL630138
				NP2 303	M	22.06.2019	OL628757	–	OL630139
			14°39′00.4″ N 98°34′33.7″ E	NP3 149	M	22.06.2019	OL628758	–	OL630140
				NP3 199	F	22.06.2019	OL628759	OL584123	OL630141
*Tropodiaptomus* sp.3	Kusuman, Sakon Nakhon, NE	Pond, T	17°19′31.5″ N 104°18′18.5″ E	KSM1 172	F	27.10.2018	OL628760	-	OL630142

Sampling site: locality name, province, geographical region; *C* central, *E* eastern, *N* northern, *NE* northeastern, *S* southern, *W* western; type of habitat: *P* permanent habitat type, *T* temporary habitat type.

### DNA extraction, amplification, and sequencing

One to three specimens (62 individuals in total) selected from each population were soaked in distilled water for 5–10 min. DNA extraction was then performed according to Garcia-Morales and Elias-Gutierrez^24^ with a modified HotSHOT protocol^25^. The extracted DNA was amplified by polymerase chain reaction (PCR).

Fragments of the mitochondrial marker 12S rRNA and of the nuclear gene Internal Transcribed Spacer 2 (ITS2) were amplified in the 62 specimens using the primer pairs and conditions described by Vecchioni et al.^26^ and White et al.^27^, respectively. In addition, a fragment of the nuclear gene 28S rRNA was amplified following the protocol described in Vecchioni et al.^26^. However, due to its conservative nature, the 28S rRNA was amplified only in a subset of specimens selected from the major clades obtained based on the “12S-ITS2 dataset” (see below). Details on primers and thermal cycles are reported in Supplementary Table S1.

After PCR, 5 μl of each PCR product were used to perform electrophoresis on 2% agarose gel, with a voltage of 90 V, for 20 min. The outcome of the electrophoresis was verified using a UV transilluminator. The samples that showed a clear, single band, with the expected weight for each gene fragment was purified using the ExoSAP-IT kit (Affymetrix USB). Sequencing was operated by Macrogen Inc. (Madrid, Spain) via an ABI 3130xL sequencer (Applied Biosystems). The same primers used for the PCRs were used for the direct sequencing of the PCR products. The quality of the resulting chromatograms was verified by measuring their “Phred score” value^28^. Among these, only the sequences that showed continuous readings of high-quality bases (QV > 20) were kept for subsequent analyses.

### Sequence alignment and phylogenetic analyses

12S and 28S chromatograms were checked and manually edited using the software Chromas v.2.6.2 (Technelysium, Pty. Ltd. 1998, Queensland, Australia). For ITS2, the forward and reverse sequences were assembled and the contig was examined through SEQUENCHER v.4.1.4 as suggested by Fontaneto et al.^29^. For ITS2, homozygotes consensus sequences were obtained directly from the contig. Heterozygotes sequences were carefully inspected and manually constructed when the chromatograms exhibited double peaks at a single position. Conversely, the two haplotypes were reconstructed using CHAMPURU v.1.0^30^ (http://jfflot.mnhn.fr/champuru/) for those sequences that showed multiple double peaks at several positions. All sequences were aligned using the Clustal W method^31^ as implemented in the software MEGAX^32^.

In order to test whether the mitochondrial and nuclear fragments could be combined for joint analyses, the incongruence length difference test (ILD)^33^ as implemented in PAUP* v. 4.0b10^34^ was used. According to Cunningham^35^, if p > 0.01, pooling the data improves the phylogenetic accuracy and thus it is admissible to merge the tested datasets into a single matrix. This condition was fulfilled both for the concatenated 12S and ITS2 sequences (p = 0.63), and for the concatenation of all the genetic markers analyzed in the frame of this study (i.e., 12S, ITS2 and 28S; p = 1). Accordingly, two combined mito-nuclear datasets (i.e., the “12S-ITS2 dataset” and the “12S-ITS2-28S dataset”) were analyzed in the frame of this work. The first dataset includes all the available novel 12S and ITS2 sequences; those individuals which were heterozygous at the ITS2 locus are here represented by two concatenated sequences indicated by different letters, bearing the alternative ITS2 haplotype. The second dataset includes a subset of the 12S and ITS2 sequences and the 28S sequences. For both combined datasets the software packages MrBayes v. 3.2.6^36^ and PhyML v. 3^37^ were used for inferring phylogenetic relationships through Bayesian inference of phylogeny (BI) and maximum likelihood (ML) analysis. As support measures for the nodes, bootstrap values (BS)^38^ were calculated with 1000 replicates in the ML trees, whereas in the BI tree the posterior probability values (PP) reported. The best evolution model for each dataset was selected in the software PartitionFinder ver. 1.0.1^39^ under the “Akaike Information Criterion” (AIC)^40^. The General Time-Reversible model of sequence evolution with a proportion of invariable sites was used for the 28S dataset (GTR + I). Instead, a GTR with a proportion of invariable sites and gamma-distributed rate variation among sites (GTR + I + Γ; nst = 6) was selected as the best evolutionary model for both the 12S and ITS2 datasets. In the BI analyses, two independent Markov chain Monte Carlo analyses were carried out for 1,000,000 generations (temp.: 0.2; default priors) with sampling every 1000 generations, the first 2,500 trees were discarded as a burn-in process and a consensus tree was constructed (Effective Sample Size (ESS) greater than 200 was reached in all the analyses performed). An individual of *Eudiaptomus intermedius* (Steuer, 1897) from a pond near the village of Sales, Italy (coordinates: 45.751845 N, 13.726236 E) was included in all the phylogenetic analyses as an outgroup. Bézier curves connecting the haplotypes found co-occurring in heterozygous individuals were added to the phylogenetic tree based on the ITS2 dataset. In addition, a haplotype network of the “28S dataset” was created with HaplowebMaker (https://eeg-ebe.github.io/HaplowebMaker/) with the "Median-Joining" method^41^.

### Species delimitation methods

In the frame of this paper, we followed the ‘unified species concept’ described by De Queiroz^42^. Accordingly, morphological similarity or identity was not considered per se sufficient evidence for conspecificity, and single-locus DNA taxonomy approaches were implemented to explore the possible presence of groups of putative species rank within each studied morphospecies^42,43^. We applied two independent methods of species delimitation based on the 12S and ITS2 datasets. The “assemble species by automatic partitioning” (ASAP)^44^ method was implemented using the online ASAP server (https://bioinfo.mnhn.fr/abi/public/asap) with the following settings: fixed seed value = − 1 (i.e., no fixed seed value was used), and simple distance. The “multiple rate Poisson Tree Processes” (mPTP)^45^ model was run through the online mPTP server (https://mptp.h-its.org/). ASAP and mPTP analyses were performed on *Tropodiaptomus* spp. sequences, with the exclusion of the outgroup.

Genetic distances were calculated within and between the *Tropodiaptomus* clades of putative species-level using the Kimura two-parameter model with pairwise deletion in MEGAX^46^.

### Ethics statement

The present study was approved by the ethics committee of Kasetsart University (approval no. ACKU61-SCI-004) for collecting the *Tropodiaptomus* specimens.

## Results

### Morphological analysis

In the frame of this study, six *Tropodiaptomus* morphospecies were identified in the 23 sampled locations in Thailand. These are *Tropodiaptomus oryzanus*, *T. vicinus*, *T.* cf. *lanaonus*, *Tropodiaptomus* sp.1, *Tropodiaptomus* sp.2, and *Tropodiaptomus* sp.3 (Table 1). Studied specimens mostly showed variation in i**)** the ornamentation of the basis and second exopod segment of adult male right P5, ii) the inner margin of the exopod in adult male left P5, and iii) the length of the process on the antepenultimate segment of adult male right antennule **(see** Table 2**)**. Moreover, Lai et al.^16^ also reported that in the inner margin of adult male left P5 exopod in *T*. *vicinus* two or three lobes might be present; however, Thai specimens consistently showed the presence of two lobes. Conversely, our *T. vicinus* specimens showed variability in the ornamentation on second exopod of adult male right P5 and length of spinous process on the antepenultimate segment of adult male right antennule.

**Table 2:** Morphology of adult males in studied Thai *Tropodiaptomus* taxa and populations.

Taxa	Clade	Code	Total of specimen (s)	Spinous process on segment-20 VS segment-21 of right antennule				Number of setae on segment-13 of left antennule	Ornamentation on right P5		Inner margin saw of first exopod of left P5
				1/2	3/4	equal	long		Basis	Exp-2	
*Tropodiaptomus vicinus*	I	SNG1	6	–	–	4	2	1	A + H	2A + H	Two deep lobes with same pattern of serrate
		SNG3	3	–	–	–	3	1	A + H	2A + H	
		KDP1	10	–	–	–	10	1	A + H	2A + H	
		TP	5	–	–	4	5	1	A + H	2A	
	II	RBG13	3	–	3	–	–	1	A + H	2A	
	III	SMR1	9	–	2	7	–	1	A + H	2A + H	
		TMP1	5	–	–	1	4	1	A + H	2A* + H	
	IV	NN04	1	–	–	1	–	1	A + H	2A	
		KPK1	3	–	3	–	–	1	A + H	A + H	
	V	KDB	6	–	–	6	–	1	A + H	A* + H	
*Tropodiaptomus* cf. *lanaonus*	VI	NER	7	–	7	–	–	1	H	A + H	Two shallow lobes with same pattern of serrate
	VII	KPK1	7	1	6	–	–	1	A + H	A + H (n = 3), 2A (n = 4)	
	VIII	NMK4	2	1	1	–	–	1	H	A + H	
	IX	NT	2	–	2	–	–	1	A + H	2A	
*Tropodiaptomus* sp.1	X	DKT1	12	–	10	2	–	1 (*n* = 10), 2 (*n* = 2)	2A + H	A	One lobe with large serrate in proximal-midle part and small serrate in middle-distal part
*Tropodiaptomus* sp.2	XI	NP2	4	–	–	–	4	1	A + H	A + H	Two lobes with same pattern of serrate
		NP3	1	–	–	–	1	1	A + H	A + H	
*Tropodiaptomus oryzanus*	XIII	KDP1	1	–	21	–	–	1	A + 3H	A	One lobe with large serrate in middle part
		SNG4	21	–	21	–	–	1	A + 3H	A	

*A* apophysis, *H* hyaline lamella

*Distal margin of exp-2 of right fifth leg with long apophysis).

**Figure 2:**
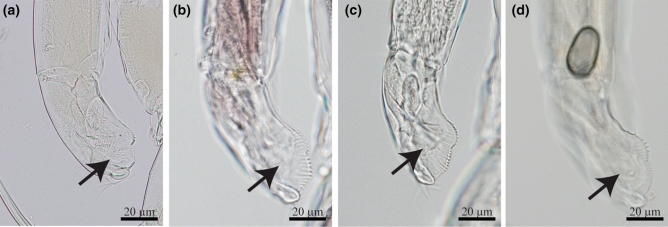
Morphology of the exopod of adult male left P5 in *Tropodiaptomus* cf. *lanaonus*. (**a**) specimen from NER. (**b**) specimen from KPK1. (**c**) specimen from NMK4. (**d**) specimen from NT.

The morphology of the specimens here ascribed to *Tropodiaptomus* cf. *lanaonus* do not agree with the original description of the species^22^ in two characters: (i) the length of the spinous process in the antepenultimate segment of adult male right antennule is longer than segment 21 in the original description, whereas in our specimens (from sites KPK1, NT, NER and NMK4, see Table 1) it is 1/2 to 3/4 of segment 21, and (ii) the ornamentation on the basis of adult male right P5 has one apophysis and one hyaline lamella in the original description, which is in accordance with Thai specimens from KPK1 and NT, whereas studied specimens from NER and NMK4 have only the hyaline lamella and no apophysis. In addition, Thai *T.* cf. *lanaonus* also showed variability in the morphology of the basis and second exopod of adult male right P5 (Table 2). Moreover, specimens from NER have a group of spinules near inner margin lobe on the exopod of adult male left P5, this character does not show in specimens from KPK, NMK4 and NT (see Fig. 2).

We refrained from ascribing to any known species the Thai *Tropodiaptomus* populations from DKT (here referred to as *Tropodiaptomus* sp.1) and NP2 and NP3 (here referred to as *Tropodiaptomus* sp.2) because the morphology of adult male P5 and right antennule in *Tropodiaptomus* sp.1 and *Tropodiaptomus* sp.2 are different from all the *Tropodiaptomus* species known to date. Moreover, *Tropodiaptomus* sp.1 and *Tropodiaptomus* sp.2 differ one from the other for the following characteristics: the basis of the right P5 of *Tropodiaptomus* sp.1 has two processes and one hyaline lamella but it has one process and one hyaline lamella in *Tropodiaptomus* sp.2; the length of the spinous process in antepenultimate segment of adult male right antennule in *Tropodiaptomus* sp.1 is 3/4 of or equal to segment 21, while it is longer than segment 21 in *Tropodiaptomus* sp.2; and the shape of second exopod of the right P5 is trapezoidal in *Tropodiaptomus* sp.1 and rectangular in *Tropodiaptomus* sp.2. Based on all these characters, we suggest that they are two new putative species pending a formal description.

In addition, a single female whose morphology was not ascribable to any known *Tropodiaptomus* species was collected from the site KSM1 and here reported as *Tropodiaptomus* sp.3. Unfortunately, no *Tropodiaptomus* males were collected from this site.

The most common morphospecies collected in the frame of present study was *Tropodiaptomus vicinus*, which was found in 13 localities; it was found co-occurring with *T.* cf. *lanaonus* (site KPK1, 13 October 2017) and *T. oryzanus* (sites KDP1 and SNG4, 3 June 2019) (Table 1). All the other *Tropodiaptomus* taxa and populations were found with no co-occurring congeneric species in each locality in this study.

### Molecular analyses

**Figure 3:**
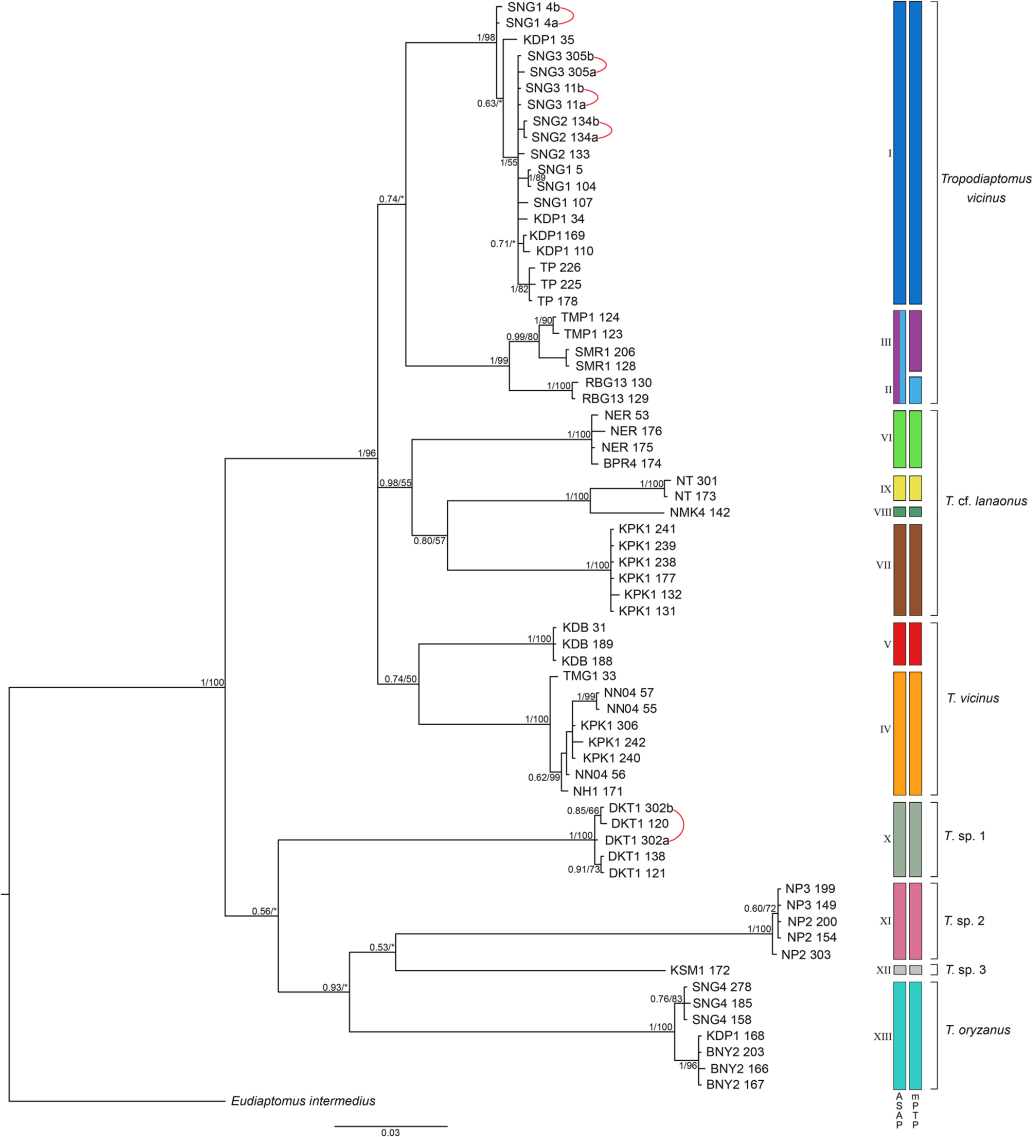
Bayesian phylogram (95% majority rule consensus tree) of *Tropodiaptomus* spp. based on the concatenated 12S-ITS2 dataset. *Eudiaptomus intermedius* was used as an outgroup to root the tree. Node statistical support is reported as nodal posterior probabilities (Bayesian Inference of phylogeny, BI)/bootstrap values (maximum likelihood, ML). Asterisks indicate support values lower than 50. Rectangles refer to MOTUs as indicated by ASAP or mPTP (see Supplementary Figs. S2, S3). Square brackets group the samples according to their morphological identification. The analyzed specimens are reported using the location and codes listed in Table 1.

Overall, a total of 62 *Tropodiaptomus* individuals were molecularly analyzed. The mitochondrial 12S and the nuclear ITS2 markers were successfully amplified in all the 62 individuals; conversely, 28S sequences were produced for a subset of 18 individuals only. See Table 1 for a synopsis of the fragments amplified for each studied specimen and their GenBank Accession Numbers. Unfortunately, no 28S amplicons could be obtained for representatives of clades VI, VIII, and XII (see Fig. 3), which are thus not represented in the analyses which include this fragment. In addition, 12S, ITS2 and 28S sequences were obtained from the Italian *Eudiaptomus intermedius* specimen used as outgroup (ANs: OL584216 for 12S, OL584119 for 28S, OL630143 for ITS2).

The phylogenetic trees (BI and ML analysis) based on the aligned 899 bp-long fragment of the concatenated “12S-ITS2 dataset” (367 bp for 12S, 533 bp for ITS2; Fig. 3) show a congruent topology, with the occurrence of 13 major clades with strong to moderate node support, hereafter indicated with roman numerals (i.e., clades I–XIII, see Fig. 3). The Thai populations ascribed to the morphospecies *Tropodiaptomus vicinus* resulted to be paraphyletic, since they were split into two major monophyletic groups with no sister-clades relationship and further subdivided in five clades. “Clade I” includes 15 specimens that belong to five different sites located in the northeastern part of Thailand (KDP1, SNG1, SNG2, SNG3 and TP; PP = 1, BS = 98%). Clade II includes two specimens from a single site located in eastern part of Thailand (RBG13; PP = 1; BS = 100%). Clade III includes four specimens from two locations in eastern and southern part of Thailand (SMR1 and TMP1; PP = 0.99, BS = 80%). Clade IV includes eight specimens from five locations in central, northeastern, and western parts (NH1, NN04, TMG1 and KPK1; PP = 1, BS = 100%). Clade V includes three specimens from one location in the northeastern part of Thailand (KDB; PP = 1, BS = 100%). All the other morphospecies proved to constitute monophyletic groups, although four well-characterized subclades are present within *Tropodiaptomus* cf. *lanaonus* (clades VI, VII, VIII, and IX, see Fig. 3).

Based on the 12S-ITS2 dataset, the genetic distance between the 13 clades reported in Fig. 3 ranged between 0.042 (*T.* cf. *lanaonus* clade VIII *vs*. *T*. cf. *lanaonus* clade IX) and 0.26 (*T. oryzanus vs. T. vicinus* clade III); the genetic distance within clades ranged between 0 and 0.013 (Table 3).

**Table 3:** Intra-clade and inter-clades genetic diversity, assessed by Kimura two-parameter distance based on the 12S rRNA-ITS2 dataset.

Taxa	Clade	n	Within Clade	Between Clade												
				I	II	III	IV	V	VI	VII	VIII	IX	X	XI	XII	XIII
*Tropodiaptomus vicinus*	**I**	19	0.005	–												
*T. vicinus*	**II**	2	0.001	0.074	–											
*T. vicinus*	**III**	4	0.013	0.095	0.046	–										
*T. vicinus*	**IV**	8	0.007	0.105	0.101	0.126	–									
*T. vicinus*	**V**	3	0.000	0.073	0.084	0.115	0.086	–								
*T.* cf. *lanaonus*	**VI**	4	0.003	0.087	0.093	0.123	0.105	0.078	–							
*T.* cf. *lanaonus*	**VII**	6	0.001	0.092	0.087	0.131	0.113	0.083	0.090	–						
*T.* cf. *lanaonus*	**VIII**	1	n/c	0.085	0.091	0.131	0.108	0.086	0.095	0.095	–					
*T.* cf. *lanaonus*	**IX**	2	0.001	0.088	0.084	0.123	0.119	0.092	0.095	0.089	**0.042**	–				
*T.* sp. 1	**X**	5	0.002	0.155	0.160	0.218	0.188	0.152	0.148	0.159	0.153	0.154	–			
*T.* sp. 2	**XI**	5	0.000	0.143	0.147	0.208	0.184	0.142	0.164	0.163	0.158	0.152	0.154	–		
*T.* sp. 3	**XII**	1	n/c	0.146	0.158	0.242	0.164	0.156	0.150	0.157	0.164	0.154	0.148	0.142	–	
*T. oryzanus*	**XIII**	7	0.007	0.196	0.200	**0.260**	0.227	0.210	0.195	0.218	0.198	0.204	0.197	0.210	0.173	–

Minimum and maximum values of genetic distance between clades are reported in bold. The presence of n/c in the results denotes cases in which it was not possible to estimate evolutionary distances.

**Figure 4:**
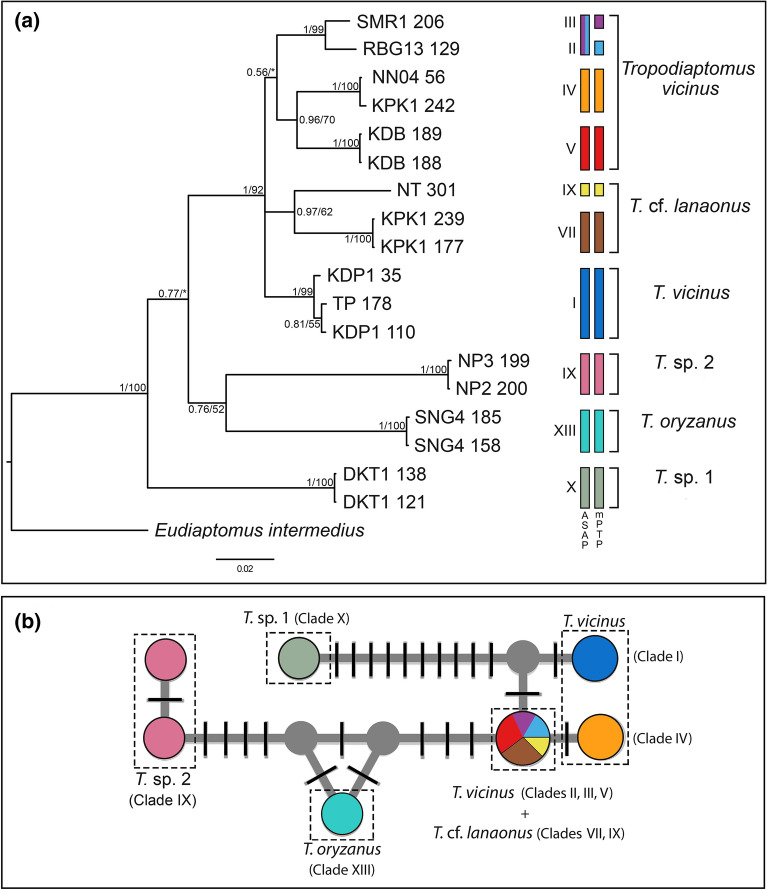
(**a**) Bayesian phylogram (95% majority rule consensus tree) of *Tropodiaptomus* spp. based on the concatenated 12S-ITS2-28S dataset. *Eudiaptomus intermedius* was used as an outgroup to root the tree. Node statistical support is reported as nodal posterior probabilities (Bayesian Inference of phylogeny, BI)/bootstrap values (maximum likelihood, ML). Asterisks indicate support values lower than 50. Rectangles refer to MOTUs as indicated by ASAP or mPTP (see Supplementary Figs. S2, S3). Square brackets group the samples according to their morphological identification. The analyzed specimens are reported using the location and codes listed in Table 1. (**b**) Median-joining haplotype network based on a fragment of the nuclear 28S ribosomal DNA. Dashes indicate substitution steps. Each circle represents a haplotype, and its size is proportional to its frequency. Dashed rectangles indicate the morphological identification of the specimens used in the analysis. Colours refer to the clades reported in (**a**).

The BI and ML phylogenetic trees based on the aligned 1709 bp-long 12S-ITS2-28S dataset (367 bp for 12S, 533 bp for ITS2, 809 bp for 28S; Fig. 4A) group the concatenated sequences in 10 major clades with moderate to strong statistical support (Fig. 4A). These ten clades are consistent with the thirteen clades singled out based on the 12S-ITS2 dataset (Fig. 3), with the exception of clades VI, VIII and XII, which are missing from this analysis due to the failure of 28S amplification (see above).

The *Tropodiaptomus* spp. haplotypes network based on the 28S nuclear marker shows the occurrence of seven haplotypes (Fig. 4B). The nine available sequences of *T. vicinus* belong to three different haplotypes. *Tropodiaptomus vicinus* specimens belonging to clades II, II and V shared the same haplotype with *T.* cf. *lanaonus* (clades VII, IX). One single haplotype was observed for *T. oryzanus* and *Tropodiaptomus* sp.1, respectively, whereas two different haplotypes were found within *Tropodiaptomus* sp.2.

### Species delimitation

**Figure 5:**
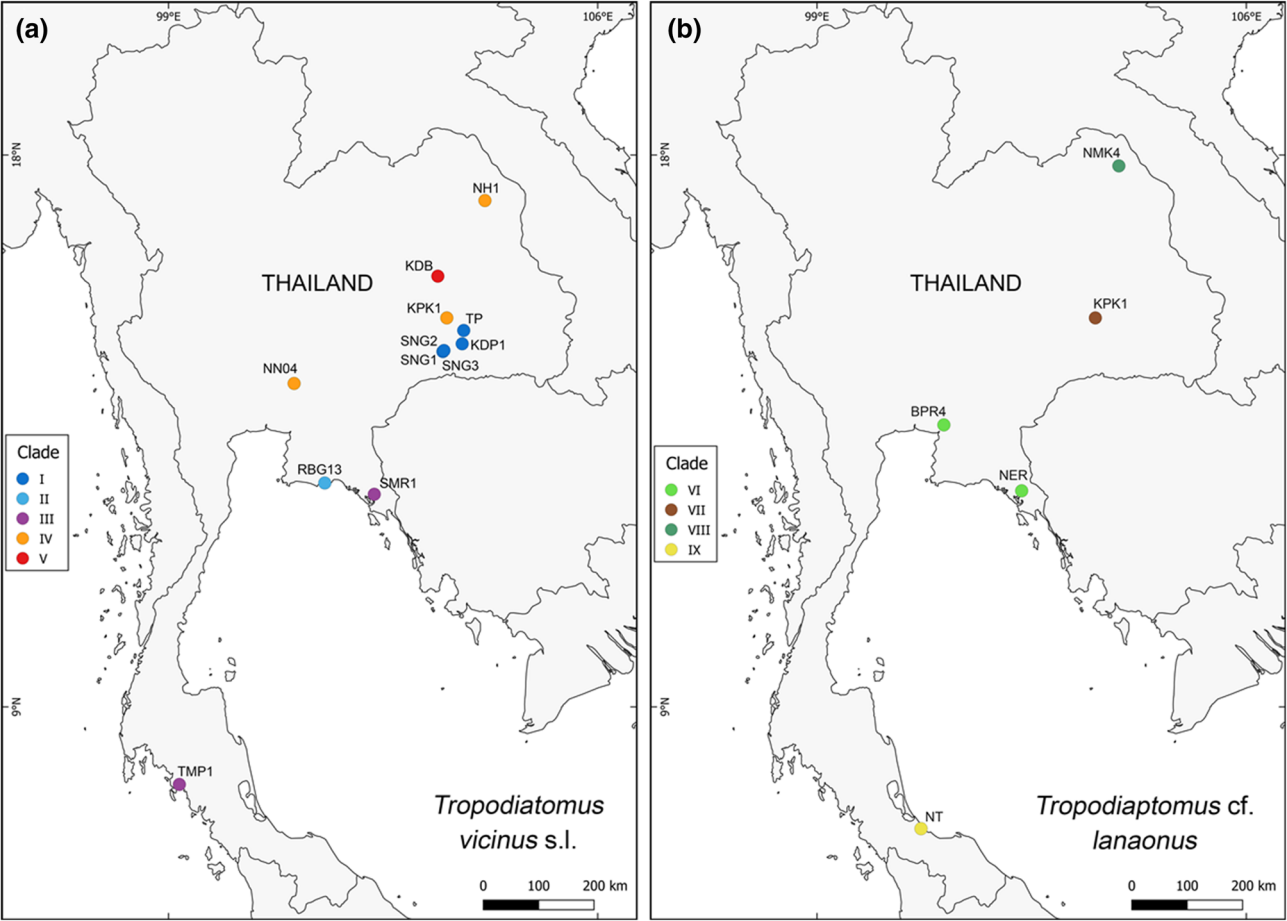
(**a**) Geographical distribution of the five clades singled out within *Tropodiaptomus vicinus* s.l. based on DNA taxonomy analyses. Colours refer to the clades reported in Fig. 3. (**b**) Geographical distribution of the four clades singled out within *Tropodiaptomus* cf. *lanaonus* based on DNA taxonomy analyses. Colours refer to the clades reported in Fig. 3. (This map was created using the QGIS software v. 3.18.3 using the layer “ne_10m_admin_0_scale_rank_minor_islands.shp” freely available at www.naturalearthdata.com/downloads/).

Based on a 365 bp-long fragment of the mitochondrial 12S gene, ASAP analysis suggested the existence of 12 groups of putative species rank within the ingroup, splitting both *Tropodiaptomus vicinus* and *T*. cf. *lanaonus* in four groups of putative species rank each; conversely mPTP analysis suggested the existence of 13 groups, splitting *Tropodiaptomus vicinus* and *T*. cf. *lanaonus* in five and four groups of putative species rank, respectively (Supplementary Figs. S1, S2, S3). The known geographical distribution of the clades found within *T. vicinus* and *T*. cf. *lanaonus* are reported in Fig. 5. Results of DNA-based classification based on the 12S gene matched morphology rather well, except for the case where putative cryptic species were found, i.e., within *T. vicinus* (clades I-V) and *T.* cf. *lanaonus* (clades VI-IX).

Based on a 533 bp-long fragment of ITS2, analyses suggested the existence of five (ASAP) or four (mPTP) groups of putative species rank, lumping in the same group specimens and populations characterized by different morphologies (Supplementary Figs. S4, S5, S6).

## Discussion

### Morphology-based species diversity of the genus *Tropodiaptomus* in Thailand

Six *Tropodiaptomus* morphospecies were found in the frame of present study, bringing to twelve the number of *Tropodiaptomus* species to date recorded in Thailand^12–14^. The species richness of *Tropodiaptomus* in Thailand is approximately 15% of the total number of species recorded for the genus worldwide. Based on morphology, *Tropodiaptomus* diversity to date recorded in Thailand is higher than that observed in other countries in Asia including India **(**9 species**)**^47^, Indonesia **(**6 species**)**^48^, Philippines **(**4 species**)**^49^, Malaysia **(**3 species**)**^15^**,** and Vietnam **(**3 species**)**^17^. Most species found in present and previous studies seem to prefer habitats characterized by a temporary hydroperiod. However, inhabited habitats might differ. For example, *T. oryzanus* was recorded from rice fields, swamps and manmade ponds in the present study but it was found also in temporary canals and ponds^50^, and roadside canals^51,52^**.**

The co-occurrence of different *Tropodiaptomus* species has been seldom observed in Thailand, and it always implied the co-occurrence of *T. vicinus* with other species, i.e., *Tropodiaptomus* cf. *lanaonus*, *T. megahyaline* and *T. oryzanus* (Table 1; see also:^13,14,51^). Conversely, *Tropodiaptomus* species are routinely observed to co-occur with other diaptomids belonging to the genera *Dentodiaptomus* Shen & Tai, 1964; *Eodiaptomus* Kiefer, 1932; *Heliodiaptomus* Kiefer, 1932; *Mongolodiaptomus* Kiefer, 1937; *Neodiaptomus* Kiefer, 1932 and *Phyllodiaptomus* Kiefer, 1936, with multi-species diaptomid coexistences ranging from two to eight species per site^13,52–54^.

Out of the six morphospecies identified in the frame of present survey, three taxa are putative species new to Science pending a formal description. The finding of such a high incidence of undescribed species suggests the existence of a significant “Linnean shortfall”^55^ affecting Thai diaptomid fauna, as also confirmed by the recent description of a new species of the genus^13^ and the report of a further new species pending a formal description by Sanoamuang and Dabseepai^14^. Current knowledge about Thai and Oriental *Tropodiaptomus* species is thus likely largely incomplete. Such a shortfall prevents from getting an exhaustive picture of the morphological and genetic diversity patterns of the genus, and of their taxonomical value.

### Comparison between morphological and molecular diversity patterns

The existence of a noteworthy morphological variability within the Philippine and Indian species belonging to the genus *Tropodiaptomus* was reported by Lai et al.^16^ and Ambedkar^18^, respectively. They concluded that both intra- and interspecific variations of adult male P5 can be observed. In the frame of the present study, a certain degree of morphological variability was observed within *Tropodiaptomus vicinus* and *Tropodiaptomus* cf. *lanaonus* only, whereas a negligible variability was observed in the other species (Table 2). The molecular analyses carried out on the studied species showed that four of the six morphospecies found in the frame of present study, i.e., *Tropodiaptomus oryzanus*, *Tropodiaptomus* sp.1, *Tropodiaptomus* sp.2, and *Tropodiaptomus* sp.3, could be consistently identified based on their morphology and DNA sequences. Conversely, the morphospecies *T. vicinus* and *T.* cf. *lanaonus* showed a high level of genetic diversity, which suggests that the resolution of traditional morphological techniques may be insufficient for correctly assess their statuses.

The branching patterns of the phylogenetic trees built upon both the “12S-ITS2” and “12S-ITS2-28S” datasets (Figs. 3, 4a) show a noteworthy structuring within these last two morphospecies, which, along with their morphological variability (Table 2) and the paraphyly of *T. vicinus* (Fig. 3), is suggestive of the possible existence of multiple taxa lumped under these binomia. The two implemented DNA taxonomy analyses based on 12S sequences suggest the occurrence of four (ASAP) and five (mPTP) groups of putative species rank within *Tropodiaptomus vicinus*, and of four groups of the same rank within *Tropodiaptomus* cf. *lanaonus* for both ASAP and mPTP (Fig. 3, Supplementary Figs. S2, S3). Interestingly, such a deep genetic structure corresponds to the high variability in morphological characters observed in these two morphospecies. Conversely, species delimitation analyses based on ITS2 sequences produced results, which are counter-intuitive and not in agreement with the morphological evidence, largely underestimating the actual diversity of the ingroup. This is likely due to the slow evolutionary rate of this marker, which does not allow to properly resolve species-level relationships within *Tropodiaptomus*.

*Tropodiaptomus vicinus* was described from India^56^, and afterwards reported for Malaysia^15^, Philippines^16^, Vietnam^17^ and India^18^. Morphological variability was observed within the studied *T. vicinus* specimens from Thailand (Table 2); however, the observed characters do not allow to consistently distinguish among the clades of putative species rank suggested by DNA taxonomy analyses (Fig. 3, Table 2), and these clades are currently impossible to be told apart based on morphology. However, no clear geographic pattern of the observed molecular diversity pattern is evident (Fig. 5a), so that the existence of a geographical cline of genetic diversity seems not to be supported. Accordingly, the DNA-based clustering of the studied populations in five clades not forming a monophyletic group is neither in agreement with their morphology nor geographical distribution.

*Tropodiaptomus lanaonus* was described as an endemic species from Lake Lanao in Philippines by Kiefer^22^. Since then, the species has not been recorded until the twenty-first century, when Sanoamuang^23^ recorded the occurrence of this species in central and northeastern Thailand, and from Laos. Lopez et al.^48^ reported that this species has not been found from samples collected in the Philippines during 2008–2015 and hypothesize that the species could be locally extinct. Therefore, until now, this species has been reported only from its type locality (Lake Lanao, Philippines), from Laos, and from the eastern and northeastern parts of Thailand^14^. In the frame of present work, a *Tropodiaptomus* species close to *T. lanaonus* was collected also in southern Thailand, but due to its morphological peculiarities, which are suggestive of a possible species-level differentiation between the studied Thai populations and *T. lanaonus* s.s., we here conservatively ascribed these populations to *Tropodiaptomus* cf. *lanaonus*. A morphological re-analysis of the populations reported as *Tropodiaptomus lanaonus* in recent Thai literature is advisable in order to compare them with the *Tropodiaptomus* cf. *lanaonus* sampled in the frame of this survey. Based on molecular data, *Tropodiaptomus* cf. *lanaonus* consists of four clades of putative species rank (Fig. 3) with an allopatric distribution (Fig. 5b) but, as also observed for *Tropodiaptomus vicinus* (see above), morphological characters do not allow to consistently distinguish among clades.

The spreading of molecular techniques and DNA taxonomy revealed that cryptic or overlooked species are an evolutionary constant also among diaptomids (e.g.^3–7,57^). The possible occurrence of cryptic, or simply overlooked, species within the widespread, but locally rare, *Tropodiaptomus vicinus* and *T*. cf. *lanaonus* is thus verisimilar and somehow expected. DNA taxonomy methods are powerful tools for the recognition of cryptic species and, according to the International Commission on Zoological Nomenclature (ICZN), “new species can be described on the basis of DNA sequences”^58^ (ICZN, 1999; accessed 27 July 2020). However, as stressed by Fontaneto et al.^29^ and Dellicour and Flot^43^, the clades of putative species rank singled out by DNA taxonomy methods must be considered just as “primary species hypotheses”, and the implementation of an integrative approach including the search for a consensus among the evidence provided by different data sources, e.g., morphology, ecology, biogeography, and ethology, must be adopted before formally proposing their actual species status.

The small inter-clades distances observed for some of the clades of putative species-level found in the frame of present survey (Table 3) suggest caution in order to avoid the risk of an unsupported oversplitting of the taxa. In particular, the possible incomplete sampling of the actual genetic diversity of the Thai populations of *Tropodiaptomus vicinus* and *T*. cf. *lanaonus* might lead to the finding of spurious inter-clades gaps, which would bias subsequent DNA taxonomy analyses (e.g.^59,60^). This is particularly evident for *Tropodiaptomus* cf. *lanaonus*, whose four clades of putative species rank correspond in fact to four clusters of geographically isolated populations (Fig. 5b), so that the occurrence of intermediate haplotypes in geographically intermediate areas, which would reveal a scenario of a clinal distribution of their genetic diversity, cannot be excluded. Moreover, even in the absence of intermediate haplotypes, the genetic differentiation of allopatric populations might be ascribable to local adaptation, genetic drift, or simple isolation by distance phenomena instead of to actual speciation processes.

Conversely, it is not likely that a wider sampling effort dedicated to *T. vicinus* would change the paraphyletic status suggested for the Thai populations of this morphospecies (Fig. 3), which seems to actually harbour a relevant cryptic diversity, as also suggested by the absence of a clear geographical pattern of molecular diversity (Fig. 5a). In fact, when genetically distinct clades observed within a morphospecies are found in sympatry or constitute a paraphyletic group, this provides strong indirect evidence that these entities are actually independent evolutionary units (cf.^9^).

Pending other studies and evidence, we thus here refrain from considering the highlighted clades within *Tropodiaptomus vicinus* and *T*. cf. *lanaonus* as taxa of species rank, at the same time stressing the urgency to an in-depth analysis of the diversity pattern observed within these two morphospecies with dedicated surveys covering the whole potential distribution area of these taxa in the Oriental region.

The apparent decoupling between morphological- and molecular-based assessments of Thai *Tropodiaptomus* diversity is here ascribed to the morphological conservatism of the studied taxa, coupled with an inadequate understanding of the taxonomical value of the characters traditionally used when describing or identifying species. Accordingly, the taxonomic diagnostic morphological characters proposed in recent keys to the genus (e.g.^13,18,22,47^) should be used with caution and coupled, when possible, with a molecular characterization of the studied populations.

### Concluding remarks

Currently knowledge of the distribution of Thai diaptomid species is largely incomplete and more dedicated studies should be realised to get a sound picture of the actual distribution of the studied taxa. This problem affecting biodiversity studies, known as “Wallacean shortfall”^55^, severely hinders our understanding of the diversity, ecology, and natural history of diaptomids. Present results thus stress that the diversity of Thai diaptomid copepods is currently largely underestimated due both to the Linnean and Wallacean shortfalls. Stabilizing selection and the focus of traditional morphological studies on a few “classical” characters along with the practical difficulties linked with sampling throughout vast and sometimes difficult-to-reach areas, might in fact have been leading to a gross underestimate of the diversity of Diaptomidae fauna of Thailand. The realization of further systematic faunal surveys in currently undersampled areas, coupled with the integrative morphological and molecular study of the collected species, is desirable before any taxonomical act involving cryptic species is done.

We hope that this work might pave the way to inspire future work aimed at a better knowledge of Thai diaptomids.

## Supplementary Information

Supplementary Figures.

Supplementary Tables.
