# Supplementary material for: Morphological and molecular diversity patterns of the genus Tropodiaptomus Kiefer, 1932 (Copepoda, Calanoida, Diaptomidae) in Thailand
Source: Sci Rep. 2022 Feb 9;12:2218. doi: 10.1038/s41598-022-06295-4 (PMC8828757; doi:10.1038/s41598-022-06295-4)

**
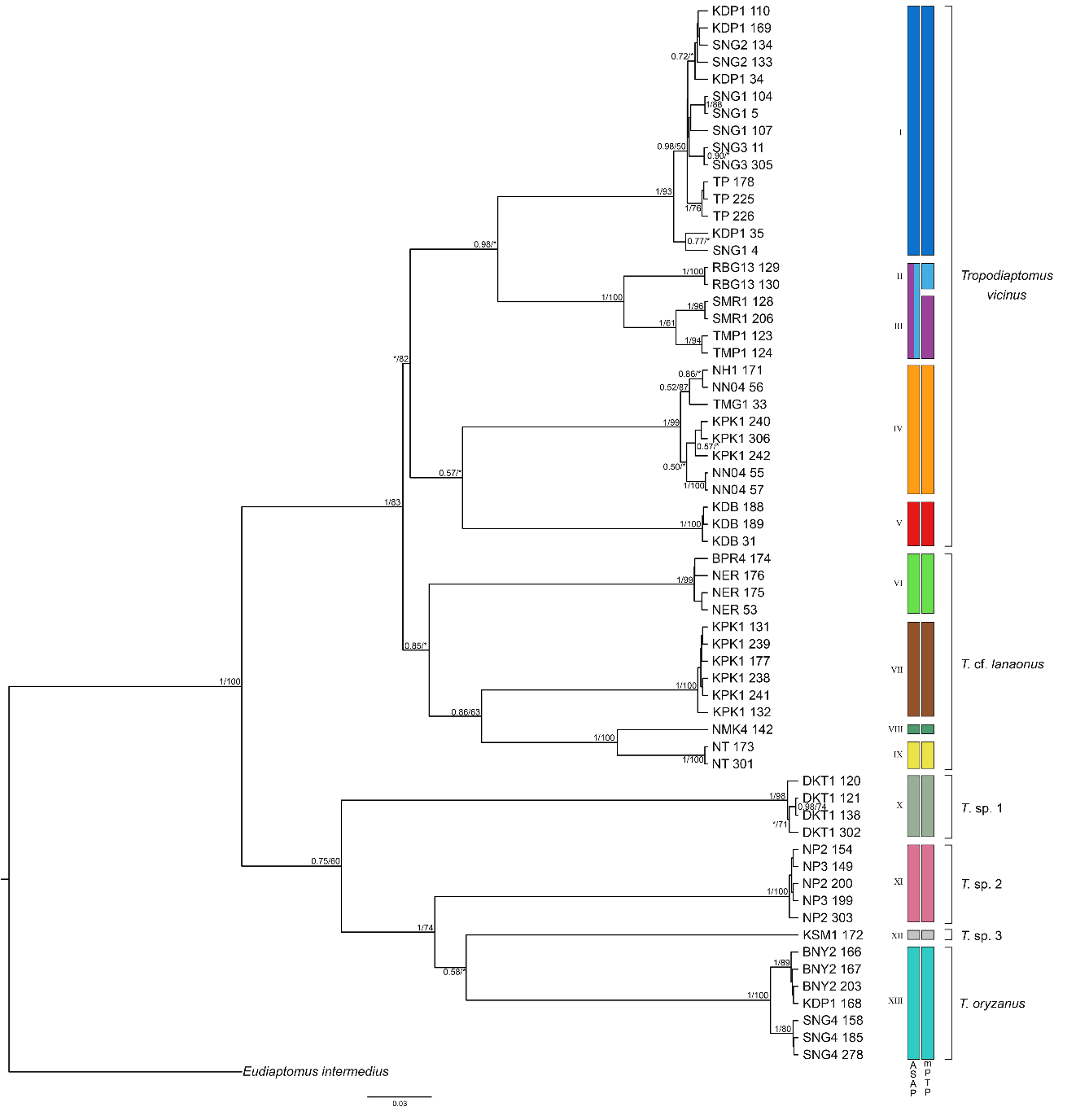
Supplementary Figure S1.** Bayesian phylogram (95% majority rule consensus tree) of *Tropodiaptomus* spp. based on the mtDNA 12S dataset. *Eudiaptomus intermedius* was used as an outgroup to root the tree. Node statistical support is reported as nodal posterior probabilities (Bayesian Inference of phylogeny, BI)/bootstrap values (maximum likelihood, ML). Asterisks indicate support values lower than 50. Rectangles refer to MOTUs as indicated by ASAP or mPTP (see Supplementary Figs. S2, S3). Square brackets group the samples according to their morphological identification. The analyzed specimens are reported using the location and codes listed in Table 1.

**Supplementary Figure S2.** Putative species singled out by ASAP based on the mtDNA 12S fragment. Codes of the analysed specimens are listed in Table 1.

**
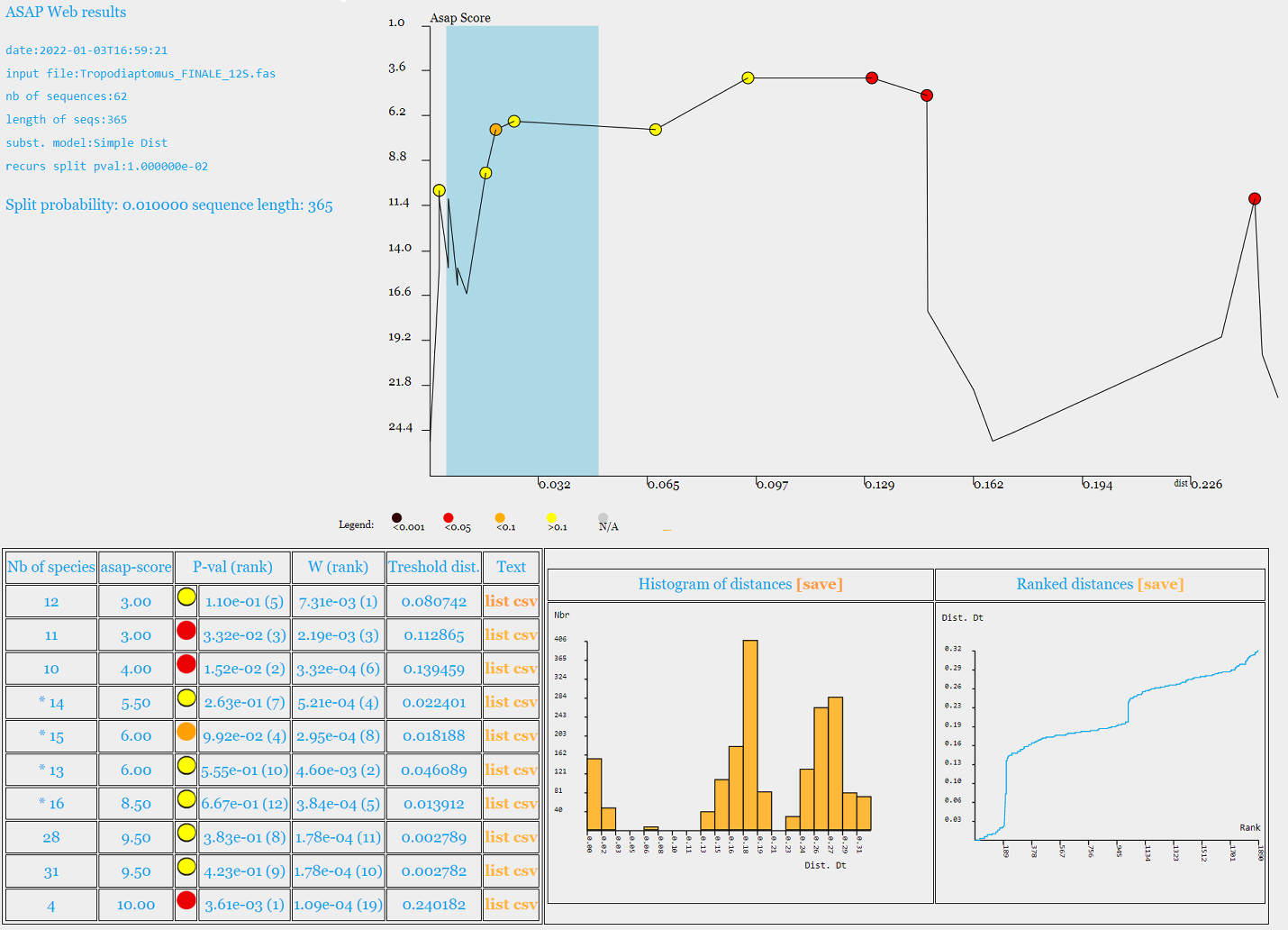
**

Number of delimited putative species: 12

**Group [1] n:6** TL_KPK1_177 TL_KPK1_238 TL_KPK1_239 TL_KPK1_241 TL_KPK1_132 TL_KPK1_131

**Group [2] n:6**  TV_TMP1_124 TV_SMR1_128 TV_SMR1_206 TV_TMP1_123

TV_RBG13_129 TV_RBG13_130

**Group [3] n:4** Tsp1_DKT1_138 Tsp1_DKT1_121

Tsp1_DKT1_120 Tsp1_DKT1_302

**Group [4] n:7**

TO_BNY2_167

TO_BNY2_166 TO_BNY2_203 TO_KDP1_168

TO_SNG4_158 TO_SNG4_278 TO_SNG4_185

**Group [5] n:2** TL_NT_301

TL_NT_173

**Group [6] n:1** TL_NMK4_142

**Group [7] n:3** TV_KDB_31

TV_KDB_188 TV_KDB_189

**Group [8] n:8**

TV_NH1_171

TV_NN04_55 TV_NN04_57 TV_TMG1_33 TV_NN04_56 TV_KPK1_242 TV_KPK1_306 TV_KPK1_240

**Group [9] n:1**

Tsp3_KSM1_172

**Group [10] n:15** TV_KDP1_169 TV_SNG2_134 TV_SNG1_4

TV_TP_178

TV_TP_225 TV_SNG1_107 TV_SNG3_305

TV_SNG3_11

TV_TP_226

TV_KDP1_35 TV_SNG1_104

TV_KDP1_110 TV_SNG2_133 TV_KDP1_34 TV_SNG1_5

**Group [11] n:5** Tsp2_NP3_149 Tsp2_NP2_303 Tsp2_NP2_200 Tsp2_NP3_199 Tsp2_NP2_154

**Group [12] n:4** TL_NER_53

TL_NER_175

TL_NER_176

TL_BPR4_174

**Supplementary Figure S3.** Putative species singled out by the mPTP model based on the mtDNA 12S fragment. Codes of the analysed specimens are listed in Table 1.

Number of edges greater than minimum branch length: 122 / 122

Null-model score: 375.705307

Best score for multi coalescent rate: 375.705307

Number of delimited putative species: 13

**Species 1:**

TL_NER_176

TL_NER_175

TL_NER_53

TL_BPR4_174

**Species 2:**

TL_KPK1_131

TL_KPK1_239

TL_KPK1_177

TL_KPK1_238

TL_KPK1_241

TL_KPK1_132

**Species 3:**

TL_NMK4_142

**Species 4:**

TL_NT_173

TL_NT_301

**Species 5:**

TV_NH1_171

TV_NN04_56

TV_TMG1_33

TV_KPK1_240

TV_KPK1_306

TV_KPK1_242

TV_NN04_55

TV_NN04_57

**Species 6:**

TV_KDB_188

TV_KDB_189

TV_KDB_31

**Species 7:**

TV_KDP1_110

TV_KDP1_169

TV_SNG2_134

TV_SNG2_133

TV_KDP1_34

TV_SNG1_104

TV_SNG1_5

TV_SNG1_107

TV_SNG3_11

TV_SNG3_305

TV_TP_178

TV_TP_225

TV_TP_226

TV_KDP1_35

TV_SNG1_4

**Species 8:**

TV_RBG13_129

TV_RBG13_130

**Species 9:**

TV_SMR1_128

TV_SMR1_206

TV_TMP1_123

TV_TMP1_124

**Species 10:**

TO_BNY2_166

TO_BNY2_167

TO_BNY2_203

TO_KDP1_168

TO_SNG4_158

TO_SNG4_185

TO_SNG4_278

**Species 11:**

Tsp3_KSM1_172

**Species 12:**

Tsp2_NP2_154

Tsp2_NP3_149

Tsp2_NP2_200

Tsp2_NP3_199

Tsp2_NP2_303

**Species 13:**

Tsp1_DKT1_120

Tsp1_DKT1_121

Tsp1_DKT1_138

Tsp1_DKT1_302

**
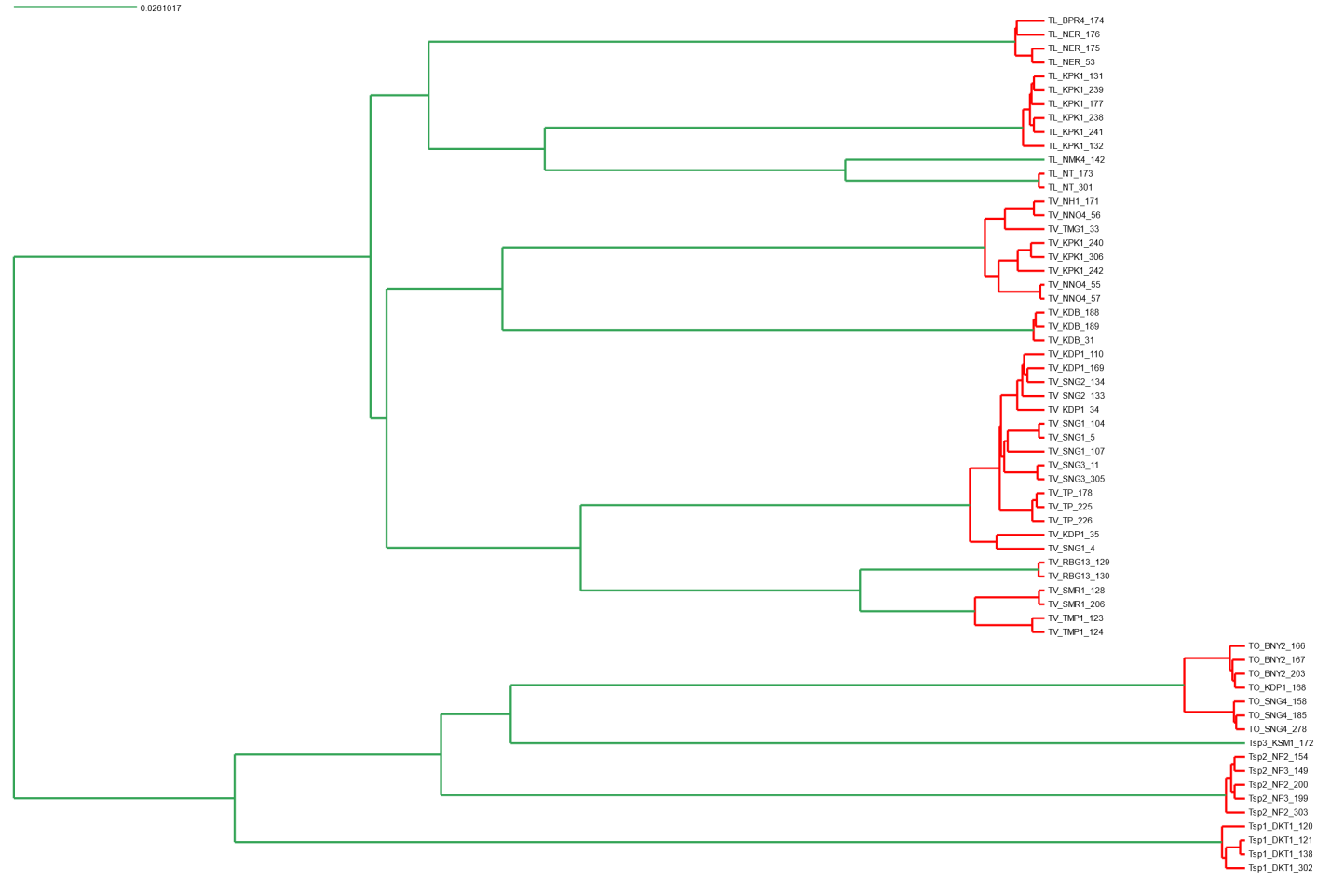
**

**Supplementary Figure S4.** Bayesian phylogram (95% majority rule consensus tree) of *Tropodiaptomus* spp. based on the nuDNA ITS2 dataset. *Eudiaptomus intermedius* was used as an outgroup to root the tree. Node statistical support is reported as nodal posterior probabilities (Bayesian Inference of phylogeny, BI)/bootstrap values (maximum likelihood, ML). Asterisks indicate support values lower than 50. Rectangles refer to MOTUs as indicated by ASAP or mPTP (see Supplementary Figs. S5, S6). Square brackets group the samples according to their morphological identification. The analyzed specimens are reported using the location and codes listed in Table 1. Colours refer to the clades reported in Figure 3 with the exception of the white rectangles, which correspond to admixed clades.


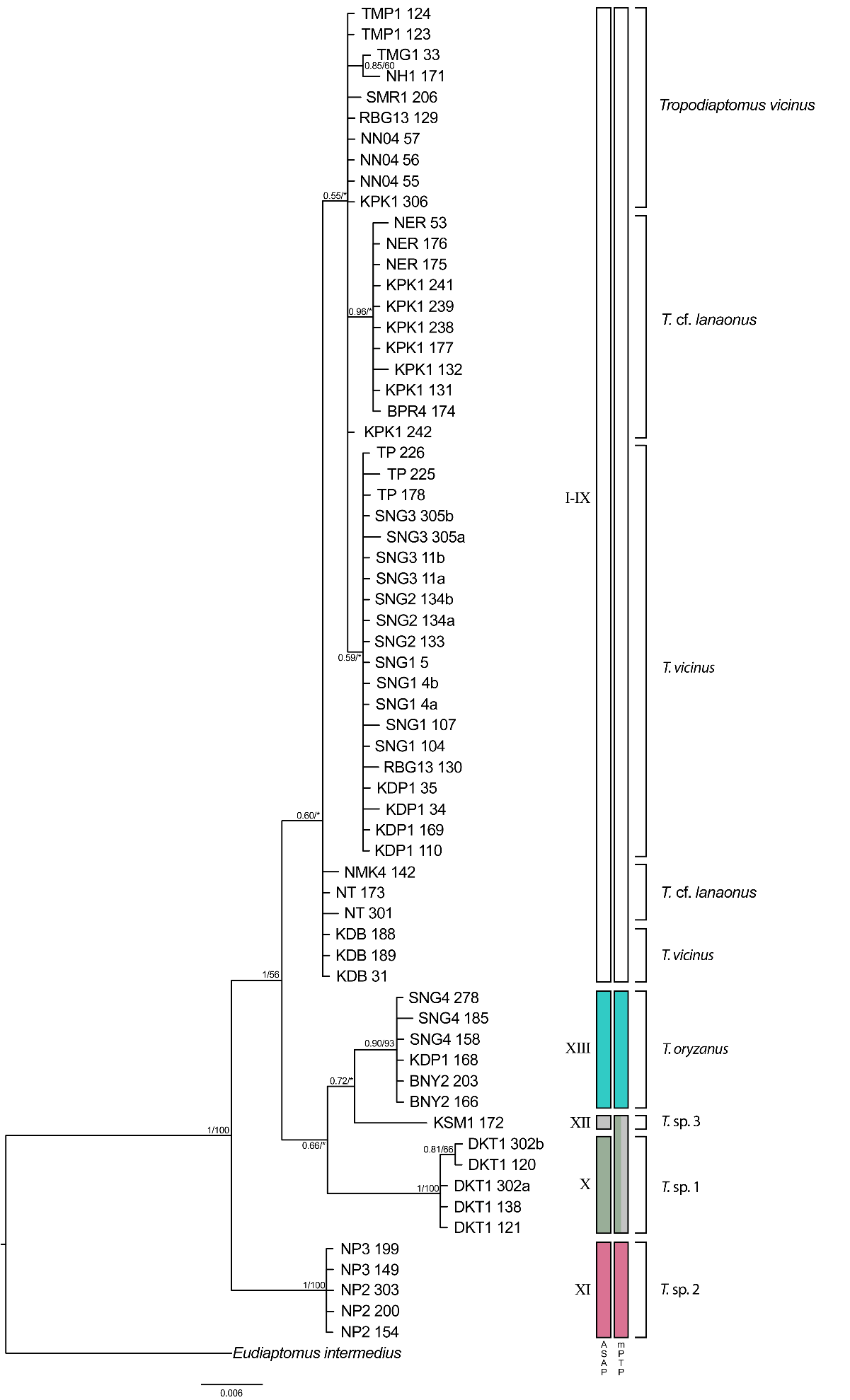


**Supplementary Figure S5.** Putative species singled out by ASAP based on the nuDNA ITS2 fragment. Codes of the analysed specimens are listed in Table 1.

**
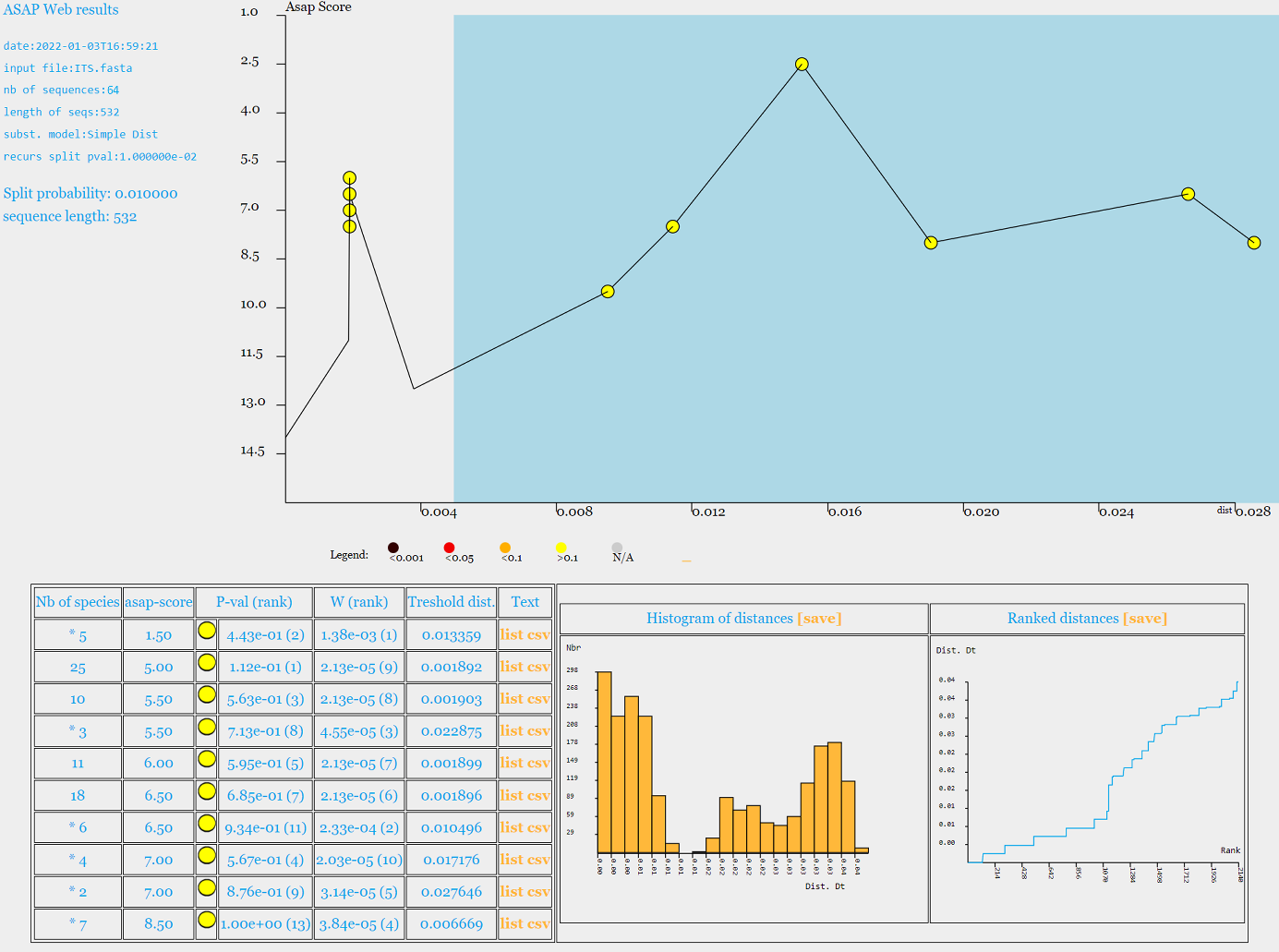
**

Number of delimited putative species: 5

**Group [1] n:47**

TV_TP_226

TV_TP_178

TV_SNG1_4_a

TV_KDP1_35

TV_KDP1_169

TV_KDP1_110

TV_SNG3_305_b

TV_SNG3_11_b

TV_SNG3_11_a

TV_SNG2_134_b

TV_SNG2_134_a

TV_SNG2_133

TV_SNG1_5

TV_SNG1_4_b

TV_SNG1_104

TV_SNG3_305_a

TV_SNG1_107

TV_TP_225

TV_TMP1_124

TV_TMP1_123

TV_RBG13_129

TV_SMR1_206

TV_RBG13_130

TV_TMG1_33

TV_NNO4_57

TV_NNO4_56

TV_NNO4_55

TV_KPK1_306

TL_KPK1_242

TV_NH1_171

TV_KDP1_34

TV_KDB_31

TV_KDB_189

TV_KDB_188

TL_NT_173

TL_NT_301

TL_NMK4_142

TL_NER_53

TL_NER_176

TL_NER_175

TL_KPK1_241

TL_KPK1_239

TL_KPK1_238

TL_KPK1_177

TL_KPK1_131

TL_BPR4_174

TL_KPK1_132

**Group [2] n:6**

TO_SNG4_278

TO_SNG4_158

TO_KDP1_168

TO_BNY2_203

TO_BNY2_166

TO_SNG4_185

**Group [3] n:5** Tsp2_NP3_199 Tsp2_NP3_149 Tsp2_NP2_303 Tsp2_NP2_200 Tsp2_NP2_154

**Group [4] n:5** Tsp1_DKT1_302_b Tsp1_DKT1_120 Tsp1_DKT1_302_a Tsp1_DKT1_138 Tsp1_DKT1_121

**Group [5] n:1**

Tsp3_KSM1_172

**Supplementary Figure S6.** Putative species singled out by the mPTP model based on the nuDNA ITS2 fragment. Codes of the analysed specimens are listed in Table 1.

Number of edges greater than minimum branch length: 30 / 126

Null-model score: 120.552901

Best score for multi coalescent rate: 120.552901

Number of delimited species: 4

**Species 1:**

TV_SNG1_4_b

TV_SNG1_104

TV_SNG1_5

TV_SNG2_133

TV_SNG2_134_a

TV_SNG2_134_b

TV_SNG3_11_a

TV_SNG3_11_b

TV_SNG3_305_a

TV_SNG1_107

TV_SNG3_305_b

TV_KDP1_34

TV_TP_225

TV_TP_226

TV_TP_178

TV_SNG1_4_a

TV_KDP1_35

TV_KDP1_169

TV_KDP1_110

TV_RBG13_130

TV_TMP1_124

TV_TMP1_123

TV_RBG13_129

TV_SMR1_206

TV_TMG1_33

TV_NH1_171

TV_NN04_57

TV_NN04_56

TV_NN04_55

TV_KPK1_306

TL_KPK1_242

TL_NMK4_142

TL_NT_301

TV_KDB_31

TV_KDB_189

TV_KDB_188

TL_NT_173

TL_KPK1_132

TL_NER_53

TL_NER_176

TL_NER_175

TL_KPK1_241

TL_KPK1_239

TL_KPK1_238

TL_KPK1_177

TL_KPK1_131

TL_BPR4_174

**Species 2:**

TO_SNG4_185

TO_SNG4_278

TO_SNG4_158

TO_KDP1_168

TO_BNY2_203

TO_BNY2_166

**Species 3:**

Tsp3_KSM1_172

Tsp1_DKT1_302_b

Tsp1_DKT1_120

Tsp1_DKT1_302_a

Tsp1_DKT1_138

Tsp1_DKT1_121

**Species 4:**

Tsp2_NP2_200

Tsp2_NP2_154

Tsp2_NP2_303

Tsp2_NP3_149

Tsp2_NP3_199


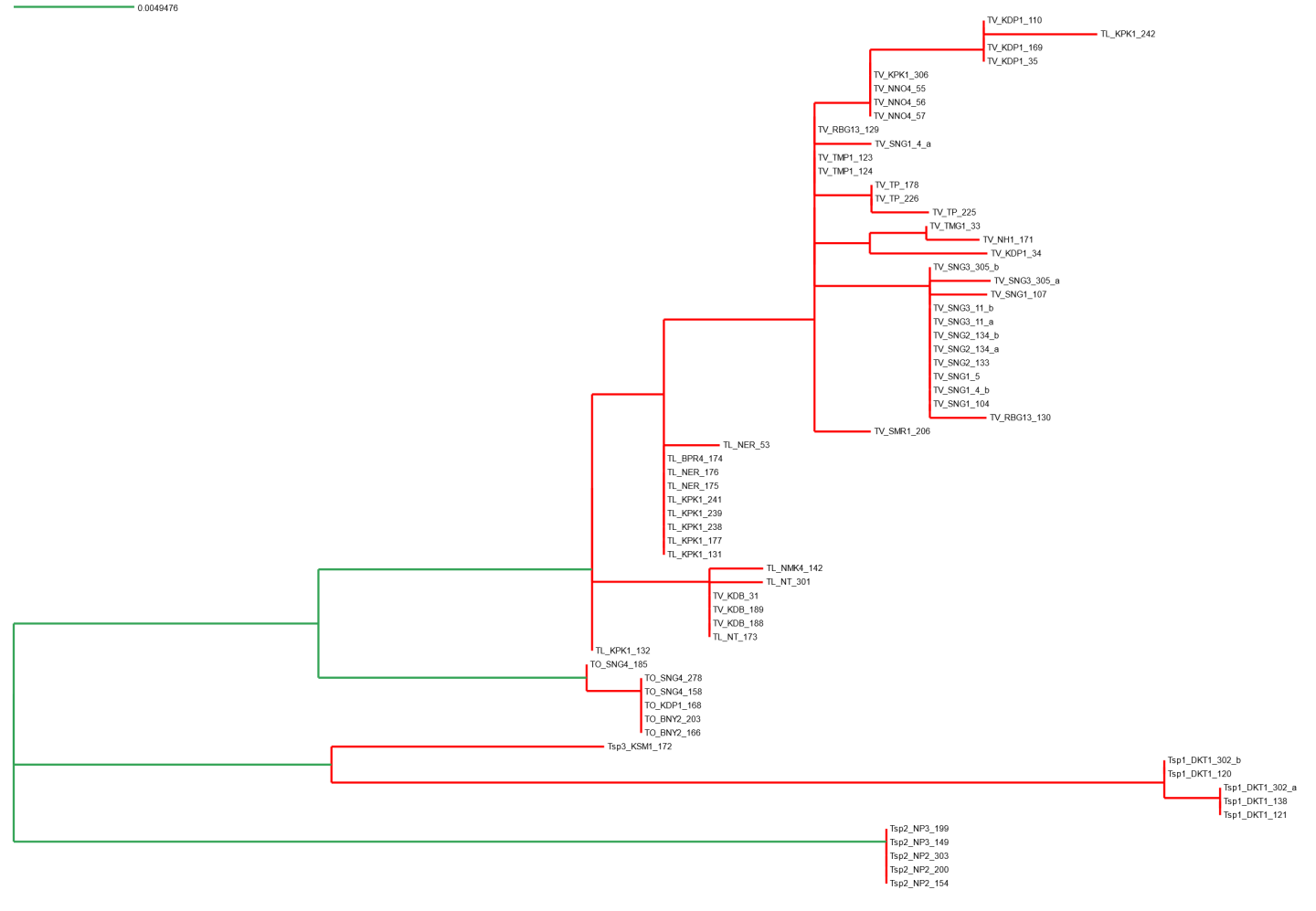

Supplement: Supplementary file 1 — Supplementary Figures. [file 41598_2022_6295_MOESM1_ESM.docx]
