# Supplementary material for: Morphological and molecular diversity patterns of the genus Tropodiaptomus Kiefer, 1932 (Copepoda, Calanoida, Diaptomidae) in Thailand
Source: Sci Rep. 2022 Feb 9;12:2218. doi: 10.1038/s41598-022-06295-4 (PMC8828757; doi:10.1038/s41598-022-06295-4)
Supplement: Supplementary file 2 — Supplementary Tables. [file 41598_2022_6295_MOESM2_ESM.docx]

**Supplementary Table S1.** Primers and thermal cycling protocols used for the amplification of the three loci used to investigate the molecular diversity of Thai *Tropodiaptomus* taxa. (* = Primer used for sequencing reaction).

| **Locus** | **Primer (5’ – 3’)** | **Primer reference** | **Thermal cycling protocol** | **Product length (analysed)** |
| --- | --- | --- | --- | --- |
| 12S | L13337: YCTACTWTGYTACGACTTATCTC* | Machida et al.^1^ | [15 s at 96 °C, 30 s at 45 °C, 15 s at 72 °C] x 30 | 367 bp |
|  | H13845: GTGCCAGCAGCTGCGGTTA |  |  |  |
| 28S | 28S-F1A: GCGGAGGAAAAGAAACTAAC* | Ortman^2^ | [60 s at 95 °C, 60 s at 48 °C, 60 s at 72 °C] x 35 | 809 bp |
|  | 28S-R1A: CGATAGTTTCACCATCTTTCGGG |  |  |  |
| ITS2 | ITS-3F: GCATCGATGAAGAACGCAGC* | White et al.^3^ | [30 s at 95 °C, 30 s at 50 °C, 60 s at 72 °C] x 35 | 533 bp |
|  | ITS-10R: TACGGGCCTATCACCCTCTACG* |  |  |  |

**References**

1. Machida, R. J., Miya, M. U., Nishida, M., & Nishida, S. Complete mitochondrial DNA sequence of *Tigriopus japonicus* (Crustacea: Copepoda). *Marine Biotechnology* **4**(4), 406–417, https://doi.org/10.1007/s1012 6-002-0033-x (2002).
2. Ortman, B. D. *DNA barcoding the Medusozoa and Ctenophora*. (University of Connecticut, Storrs, 2008).
3. White, T. J., Bruns, T., Lee, S. & Taylor, J. W. Amplification and direct sequencing of fungal ribosomal RNA genes for phylogenetics. in *PCR protocols: a guide to methods and applications* (eds. Innis, M.A., Gelfand, D.H. Sninsky, J.J. & White, T.J.) 315–322 (Academic Press, Inc., New York, 1990).
